# Improvement of Caciotta-like cheese nutritional value by means of enrichment with blackcurrant (*Ribes nigrum*) and Cornelian cherry (*Cornus mas*)

**DOI:** 10.3389/fnut.2022.1023490

**Published:** 2023-02-10

**Authors:** Jonas Andersen, Maddalena Bosetti, Andrea Mancini, Pavel Solovyev, Tiziana Nardin, Luana Bontempo, Roberto Larcher, Elena Franciosi

**Affiliations:** ^1^Research and Innovation Centre, Fondazione Edmund Mach (FEM), San Michele all'Adige, Italy; ^2^Technology Transfer Centre, Fondazione Edmund Mach (FEM), San Michele all'Adige, Italy

**Keywords:** polyphenols, Cornelian cherry (*Cornus mas* L.), blackcurrant (*Ribes nigrum*), Caciotta-like cheese, NMR profile

## Abstract

**Introduction:**

In this study, we supplemented models of Caciotta-like cheese with blackcurrant (*Ribes nigrum*) and Cornelian cherry (*Cornus mas*), as they have a high content of polyphenols, known as phytochemicals associated with health benefits. We evaluated the microbial composition, organoleptic aspects, total phenolic content, and chemical composition of model cheeses enriched with blackcurrant and Cornelian cherry.

**Methods:**

Two different suppliers have been tested: a conventional and an organic one. Two different conditions of preparation (freeze-dried and not freeze-dried) were tested in two different amounts (0.3 and 0.6% dry weight w/v milk volume). Polyphenols were determined using Folin–Ciocalteu reaction and spectrometry; microbial community was determined with selective 24 media and plate counts; composition was determined using nuclear magnetic resonance spectrometry. Organoleptic tests with an untrained panel have been performed.

**Results:**

The enrichments with blackcurrant and Cornelian cherry increased the total polyphenol content in model cheeses, in particular, when blackcurrant and Cornelian cherry were from conventional farming. Blackcurrant-enriched cheeses showed higher counts of lactic acid bacteria, higher levels of organic acids, amino acids, gamma-aminobutyric acid, histamine, and lower amount of monosaccharides deriving from bacterial lactose fermentation in cheese, suggesting a positive effect of blackcurrant compounds on the growth and activity of lactic acid bacteria. The enrichments did not affect the acceptance of the cheese, neither by blackcurrant nor by Cornelian cherry incorporation, with the exception of the appearance.

**Discussion:**

Overall, we showed that cheeses enriched with blackcurrant or Cornelian cherry from conventional farming increased the bioactive potential of the dairy product without having an adverse effect on the microbial community, physiochemical properties, or organoleptic properties.

## Introduction

Polyphenols are a large family of phytochemicals, which are interesting due to their potential beneficial bioactivity. Although in plants they mainly serve as protective compounds (1), for humans, they have been shown to be neuroprotective (2–4), anti-inflammatory (5–7), anti-carcinogenic (8, 9), and antidiabetic (10, 11), as well as having beneficial cardiovascular (12–16) and gastrointestinal effects (5, 6, 17–19). Polyphenols exert their bioactivity through direct or indirect reduction of oxidative stress (12, 13, 15, 20–23), as well as through regulating gene expression (24), activation of proteins (16, 25–27), cell signaling (4, 7), and modulating the gut microbiome (17–19). Bioactive polyphenols are found in a wide variety of plant-based products, such as tea, herbs, fruits, and vegetables, and one way to exploit their potential is by adding them as enrichment for ingredients in food manufacturing.

Enrichment is a procedure where the nutritional or bioactive potential of a food product is improved by the addition of one or more ingredients, either micronutrients (28), natural products (29), foodstuff (30, 31), by-products (32), and/or microorganisms (33).

Caciotta cheese is a semi-soft cheese with a short to medium ripening time (commonly from 15 days to 1 month), weighing about 1 kg and produced from pasteurized whole cow milk alone (34). Nowadays, there is a growing consumer interest in health-enhancing foods, and in particular, health-enhancing dairy food, whose acceptance by consumers is very high (35). The development of dairy products with added natural supplements provides a natural appeal to these foods. These supplements, besides being generally recognized as safe, confer flavor and color to dairy foods (36). In this aspect, blackcurrant (*Ribes nigrum*) (37) and Cornelian cherry (*Cornus mas*) (38, 39) have characteristic flavor, intense blue and red colors, and high content of bioactive polyphenols, and have been shown to increase phenolic contents and antioxidant activity when used in dairy products enrichment (40–42).

Previous studies reported cheese and dairy products enrichment with fruits and herbs (30, 31, 43, 44), plant extracts (45), and purified phenolic compounds (46), with an evident increase in bioactive phenolic content and antioxidant activity and improved nutritional properties. In this study, we enriched a Caciotta model cheese with polyphenols through the addition of blackcurrant and Cornelian cherry. We tested both organic and conventional suppliers, as it has been shown that organic plants, and in particular organic blackcurrant, can carry more polyphenols than conventionally farmed plants, probably due to the defense effect of these compounds against pathogens (47). We also investigated if the freeze-drying technique of the blackcurrant or Cornelian cherry could affect the final total amount of polyphenols in cheese because in a previous study it was shown that the drying technique could impact the bioactive compounds that we aimed to analyze (48). The microbial community was also investigated because polyphenols are known to have potent antimicrobial activities (49), and lactic acid bacteria are the basic components of the starter used in cheese-making and essential for the fermentation of cheese (50–53). Moreover, we analyzed the effect of dairy enrichment on the number of bioactive compounds, physiochemical properties, and organoleptic attributes of the model cheeses.

## Materials and methods

### Material preparation

Organic Cornelian cherries (CC) and blackcurrant berries (BB) were supplied from a local organic farm (Le Delizie del Monte Baldo, Brentonico, TN, Italy) in the Trentino Province of North Italy; the non-organic CC and BB were supplied by a different local farm of Trentino Province (Azienda Agricola Sant'Antonio, Comano Terme, TN, Italy). Both the BB and CC have been supplied and already washed, and the CC were already pitted. Both the BB and CC both were kept at 4°C in our laboratory until use.

### Freeze-drying

To freeze-dry plant material, BB and CC were prepared by weighing about 200 g that were stored at −80°C overnight and were finely ground using a grinder (Moulinex^®^, France) to achieve a standard size of particles of about 1.0 mm. The powders were stored at −80°C.

The freeze-drying process was conducted in a freeze-dryer (VirTis, Benchtop K, USA) at −80°C and between 0.1 and 0.2 mmHg for 72 ± 1 h. The dried samples were then ground using mortar and pestle and stored in polyethylene falcon at −80°C, protected from sunlight until further analysis or use.

Regarding the freeze-drying of cheese, the cheese samples were prepared by weighing about 10 g and then grating before the freeze-drying process. The freeze-drying process was performed in aluminum pans; samples were pre-frozen in liquid nitrogen for 3 min and then placed on the freeze-dryer trays (thickness of 1 cm).

### Experimental design

**Figure 1:**
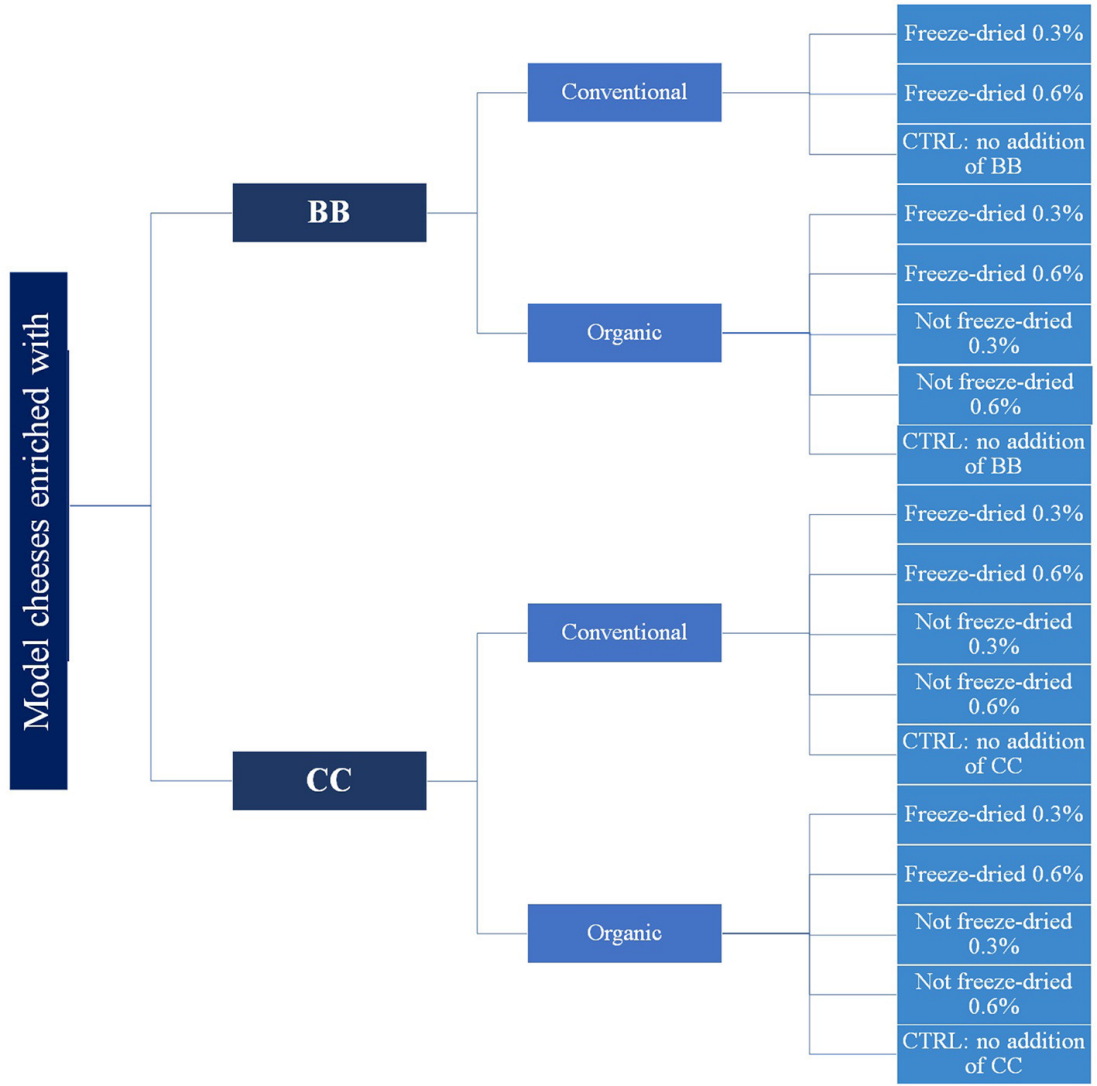
Experimental design of cheese manufacturing. Four different conditions were tested in cheese samples: conventional freeze-dried berries, organic freeze-dried berries, conventional not freeze-dried berries, and organic not freeze-dried berries. For each of these conditions, two different percentages of berries were added: 0.3 and 0.6% dry weight w/v milk volume. A cheese without the addition of berries was processed for each trial (CTRL). All the experimental conditions were carried out in triplicates.

The experimental design is shown in Figure 1. For CC, four different conditions were tested: conventional freeze-dried CC, organic freeze-dried CC, conventional not freeze-dried CC, and organic not freeze-dried CC. For BB, the conventional not freeze-dried condition was missing because there was not enough blackcurrant from the conventional farm. All the experimental conditions were carried out in triplicate samples for a total of 54 experimental model kinds of cheese (from here on out, “model cheeses” will be called only “cheeses” to simplify the reading). For each of the four conditions, two different percentages of CC and BB were added: 0.3 and 0.6% dry weight w/v milk volume. The weight was always referred to the fresh CC and BB before freeze-drying. For each trial, a control cheese (CTRL) was made without adding CC or BB.

### Caciotta-like cheese manufacture

Pasteurized whole cow milk was supplied from a local company in Trentino Province close to the lab (Abbasciano Giuseppe and C. S.N.C., Trento, TN, Italy). The milk was collected in tanks and immediately transported to the laboratory for cheese-making.

Experimental cheeses were manufactured according to the method described by Cipolat-Gotet et al. (54), with some modifications in order to obtain a Caciotta-like cheese. Milk (2 L in each vat) was heated at 40°C and inoculated with starter cultures following supplier instructions (LYOFAST MOT 086 EE, Sacco, Cadorago, CO, Italy). The curd was obtained in 10–15 min after the addition of 2 mL of liquid calf rennet (Caglificio Clerici, Cadorago, CO, Italy). To verify that the milk was perfectly curdled due to casein coagulation, a spoon was placed on the surface of the resting milk. When the spoon come out completely clean, the curd was cut into nut-size grain squares and cooked at 42°C for 10 min. The curd was incubated in the vat for 15 min and then separated from the whey. Finally, CC and BB powders were added according to the experimental design. After molding, the experimental cheeses were pressed again and turned every 30 min in a cabinet at 28°C in order to keep the curd warm. After overnight incubation at 28°C, cheeses were salted for 30 min in a 20% NaCl brine solution at room temperature, then ripened for 1 week at 18°C and finally for 4 weeks at 5°C. The pH and temperature were monitored at six different steps: (i) milk, (ii) after the addition of the starter culture, (iii) after the addition of the rennet, (iv) after the cooking, (v) the curdle at the extraction, and (iv) after 24 h of cheese incubation at 28°C.

### Microbiological analysis

Cheese samples were submitted for microbiological analysis. Four grams of cheese were homogenized with 36 g of sterile Na-citrate 2% (w/w) solution by ULTRA-TURRAX^®^ (IKA^®^ Werke GmbH and Co. KG, Staufen, Germany) for 5 min at 21,000 rpm inside the microbial cabinet. Then, they were decimally diluted and plated onto selective agar media and incubated as follows: M17 agar for 48 h in anaerobiosis at 30°C and 45°C for mesophilic and thermophilic cocci-shaped LAB, respectively; MRS agar for 48 h in anaerobiosis at 45°C for thermophilic rod-shaped LAB; and violet red bile agar (VRBA) for 24 h in anaerobiosis at 37°C for coliforms.

Berries were also submitted for microbiological analysis: 2 g of berries were homogenized with 18 g of sterile Na-citrate 2% (w/w) solution, then homogenized, diluted, plated onto selective agar media, and incubated as follows: principal component analysis (PCoA) with skim milk (10 g/L, w/v) for 48 h in aerobiosis at 30°C for total bacterial aerobic count (TBAC), WL agar with chloramphenicol (0.1 g/L, w/v) in aerobiosis at 25°C for 2 days for yeast count, and 5 days for molds count.

All culture media were purchased from Oxoid (ThremoFisher, Milan, Italy).

### Total polyphenols determination in *R. nigrum* and *C. mas* berries and cheeses

Cheese samples were grated and lyophilized. Polyphenols were extracted from each sample in triplicates of 500 mg and mixed into 12.5 mL of acidified 70/30 acetone–water solution. The mixture was homogenized, incubated at 40°C for 10 min, sonicated for 10 min, and shaken thoroughly. The samples were centrifuged at 4,000 rpm for 10 min, transferring the supernatant into 12.5 mL 96% ethanol, and the mixture was left to precipitate for 1 h at −20°C, followed by centrifugation and transfer of the supernatant. Extracted polyphenols were measured using Folin–Ciocalteu reaction. Folin–Ciocalteu (FC) colorimetric method is based on a chemical reduction of the reagent, consisting of a mixture of molybdenum oxides and tungsten (55). It represents one of the standard procedures in wine analysis, but it can also be applied to foodstuffs, such as fruits and vegetables. Total polyphenol content (TPC) is reported as the gallic acid equivalent (GAE), calculated from a standard curve previously constructed in a range of 20–200 mg/L gallic acid. BB and CC were analyzed for TPC weighing 125 mg of fresh sample, following the same extraction of the cheese. Before the Folin–Ciocalteu reaction, samples were diluted 100 times.

### Nuclear magnetic resonance spectroscopy analysis

We utilized NMR by a method modified from Rodrigues et al. (56). Samples for lipid profile analysis were obtained by thoroughly mixing 100 mg lyophilized cheese samples with 900 μL deuterated chloroform (CDCl3), and then each sample was filtered through 0.22 μm filters and 600 μL was loaded into 5 mm NMR tubes. Aliquots of 100 mg lyophilized cheese samples were mixed thoroughly with 900 μL de-ionized water (18.2 MΩ cm, Milli-Q water, Millipore, Bedford, MA, USA) and 100 μL deuterated water (D_2_O, 99.9% isotopic purity containing 0.03% 3-(Trimethylsilyl)propionic-2,2,3,3-d4 acid sodium salt or TMSP-d4). Samples were centrifuged for 15 min at 12,000 rpm. The supernatant was filtered using 0.22 μm filters (Millex-GV, polyvinylidene fluoride membrane, Millipore, Bedford, MA, USA), and 600 μL filtrate was loaded into 5 mm NMR tubes.

Nuclear Magnetic Resonance spectra were recorded on Bruker Avance Neo 7 probe (5 mm sample tubes) and SampleXpress 60-position autosampler (Bruker BioSpin GmbH, Rheinstetten, Germany). The spectra were acquired and processed using Topspin 4.1.3 software in the automation mode with Icon NMR 5.2.3. The deuterium lock signal was optimized either for the 9:1 mixture of H_2_O and D_2_O (v/v) or CDCl_3_. All proton NMR spectra were recorded using the following parameters: (a) for aqueous extracts, noesygppr1d pulse sequence with automatic adjustment of water signal suppression frequency (o1p) was used and power level utilized for pulse was 47.10 dB (25 Hz suppression window), the size of the spectrum (sweep width, SW) was 13.88 ppm, time domain (TD) consisted of 65536 (64K) data points, number of scans (NS) was 128 and the number of dummy scans (DS) was 2, the time for relaxation delay (D1) was 10 s, receiver gain (RG) for all spectra was fixed at 2.25, and baseopt digitization mode was used; and (b) for lipid extracts, the zg pulse sequence was used, the size of the spectrum (sweep width, SW) was 20.48 ppm, time domain (TD) consisted of 65,536 (64K) data points, number of scans (NS) was 64 and the number of dummy scans (DS) was 4, the time for relaxation delay (D1) was 10 s, the time for relaxation delay (D1) was 10 s, receiver gain (RG) for all spectra was fixed at 4, and baseopt digitization mode was used.

Acquisition of each spectrum was preceded by automatic adjustment of the probe (ATMA routine) and automatic shimming (TOPSHIM). Spectra were processed in the TopSpin software with the size of the real spectrum (SI) set to 131072 (128K, 2xTD) data points and apk0.noe phase correction au program was applied automatically to each spectrum.

Quantitative analysis was performed using AssureNMR software (57) by an external standard method utilizing the so-called ERETIC technique (an electronic reference to access *in vivo* concentrations, (58), which is in turn based on the principle called PULCON (pulse length-based concentration determination, (59). Two liquid samples dissolved in the same solvent were measured: a compound of known concentration (2 mmol sucrose solution in water) and a sample of cheese extract using the same experimental parameters.

Precision/intermediate repeatability of the method was carried out by periodically measuring one manufacturer standard (2 mmol sucrose in water) against another (20 mmol sucrose and hippuric acid in water), again using the same experimental parameters. The accuracy of the external standard method is described as about 95% (60).

Identification of metabolites was performed in automation mode in AssureNMR utilizing the Human Metabolome Database (HMDB) (61) and the BBIOREFCODE database of NMR metabolites (v.2.01, Bruker BioSpin GmbH, Rheinstetten, Germany).

### Organoleptic evaluation

To examine the sensory properties, attributes, such as color, odor, taste, and texture, were measured according to a 7-point hedonic scale (62) varying from 10 (like extremely) to 4 (dislike extremely). Sensory attributes were evaluated by 10 experienced panelists consisting of students and academic staff from the Food Quality Department at Edmund Mach Foundation. The panelists were all familiar with these models of Caciotta-like cheese and their characteristics. Samples from each formulation were coded with random digits and were presented to the sensory panel.

### Statistical analysis

The study was carried out on a completely randomized design basis with a 3 × 2 × 2 factorial arrangement: two types of ingredients (blackcurrant and Cornelian cherry), two farming systems (organic and conventional), and two ingredient treatments (freeze-dried and not freeze-dried). The differences between these groups were evaluated using ANOVA and *post-hoc* Tukey HSD tests. We used a 95% confidence interval and a significance level of *P* < 0.05. The statistical analysis was performed using R software version 4.2.0 (https://www.r-project.org/).

For NMR metabolites, a data matrix, in which the columns are normalized spectral integral values, was obtained. The matrix was imported into the R software version 4.2.0 and subjected to the Principal Components Analysis (PCoA). Variables were shifted to be zero-centered and were scaled to have unit variance before the analysis took place.

## Results

### The pH of experimental cheeses

The pH evolution was recorded during the whole process of cheese manufacturing: in milk, after starter culture addition, after rennet addition, after cooking, at the extraction of the curdle, and after 24 h of cheese incubation at 28°C (Table 1). The pasteurized milk used for BB cheeses had a pH ranging between 7.14 and 7.26; then during cheese processing, it decreased to 6.60–6.69 after cooking and to 6.18–6.67 in the curdle at the extraction. In all BB cheeses, the pH ranged between 5.15 and 5.54 after 24 h of incubation.

**Table 1:** pH during model cheese processing.

	**Amount (%)**	**Farming system**	**Vat milk**	**Starter addition**	**Rennet addition**	**Cooking**	**Curdle**	**After 24 h ripening**
**Blackcurrant berries**								
CTRL-BB	0	-	7.26 ± 0.73^A^	6.61 ± 0.28^A^	6.65 ± 0.26^A^	6.64 ± 0.30^A^	6.67 ± 0.17^A^	5.28 ± 0.17^A^
FD-BB	0.3	Organic	7.14 ± 0.83^A^	6.65 ± 0.22^A^	6.68 ± 0.19^A^	6.68 ± 0.23^A^	6.61 ± 0.06^A^	5.24 ±0.26^A^
FD-BB	0.6	Organic	7.14 ± 0.83^A^	6.65 ± 0.24^A^	6.69 ± 0.19^A^	6.69 ± 0.19^A^	6.54 ± 0.02^AB^	5.15 ±0.15^A^
NFD-BB	0.3	Organic	7.14 ± 0.83^A^	6.61 ± 0.25^A^	6.68 ± 0.18^A^	6.64 ± 0.25^A^	6.55 ± 0.03^AB^	5.15 ±0.16^A^
NFD-BB	0.6	Organic	7.24 ± 0.56^A^	6.58 ± 0.24^A^	6.64 ± 0.22^A^	6.61 ± 0.28^A^	6.34 ± 0.12^B^	5.22 ±0.09^A^
FD-BB	0.3	conventional	7.24 ± 0.56^A^	6.59 ± 0.25^A^	6.56 ± 0.27^A^	6.60 ± 0.26^A^	6.40 ± 0.03^B^	5.54 ± 0.19^B^
FD-BB	0.6	conventional	7.24 ± 0.56^A^	6.59 ± 0.24^A^	6.57 ± 0.27^A^	6.60 ± 0.25^A^	6.18 ± 0.15^C^	5.47 ± 0.15^B^
**Cornelian cherry**								
CTRL-CC	0	-	6.70 ± 0.10^A^	6.51 ± 0.06^A^	6.47 ± 0.05^A^	6.46 ± 0.10^A^	6.57 ± 0.04^A^	5.70 ± 0.10^A^
FD-CC	0.3	Organic	6.72 ± 0.10^A^	6.52 ± 0.03^A^	6.51 ± 0.04^A^	6.53 ± 0.04^A^	6.55 ± 0.03^A^	5.48 ±0.22^B^
FD-CC	0.6	Organic	6.72 ± 0.10^A^	6.52 ± 0.03^A^	6.50 ± 0.04^A^	6.52 ± 0.03^A^	6.50 ± 0.05^AB^	5.36 ±0.23^B^
NFD-CC	0.3	Organic	6.72 ± 0.10	6.52 ± 0.03^A^	6.53 ± 0.07^A^	6.42 ± 0.08^A^	6.45 ± 0.06^AB^	5.48 ±0.26^B^
NFD-CC	0.6	Organic	6.72 ± 0.10^A^	6.51 ± 0.03^A^	6.51 ± 0.04^A^	6.42 ± 0.08^A^	6.46 ± 0.03^AB^	5.38 ±0.21^B^
FD-CC	0.3	Conventional	6.69 ± 0.11^A^	6.48 ± 0.07^A^	6.46 ± 0.06^A^	6.44 ± 0.03^A^	6.48 ± 0.09^AB^	5.70 ± 0.04^A^
FD-CC	0.6	Conventional	6.69 ± 0.11^A^	6.48 ± 0.07^A^	6.44 ± 0.06^A^	6.42 ± 0.07^A^	6.45 ± 0.04^B^	5.75 ± 0.20^A^
NFD-CC	0.3	Conventional	6.69 ± 0.11^A^	6.48 ± 0.07^A^	6.45 ± 0.07^A^	6.43 ± 0.07^A^	6.49 ± 0.08^AB^	5.79 ± 0.06^A^
NFD-CC	0.6	Conventional	6.69 ± 0.11^A^	6.48 ± 0.07^A^	6.44 ± 0.06^A^	6.45 ± 0.05^A^	6.39 ± 0.10^B^	5.75 ± 0.11^A^

For each column and each enrichment (blackcurrant and cornelian cherry), pH values with A, B, and C superscripts are significantly different (p < 0.05, one-way ANOVA with post-hoc Tukey HSD).

FD, freeze-dried; NFD, not freeze-dried; CTRL, Control; BB, blackcurrant berry; CC, cornelian cherry.

pH values were recorded in vat milk; after the addition of the starter culture; after the addition of the calf rennet; after the cooking; in the curdle at the extraction; and after 24 h of cheese ripening at 28°C.

Regarding trials with CC enrichment, all the milk batches were characterized by a pH ranging between 6.69 and 6.72, which slightly decreased to 6.42–6.53 after cooking and to 6.39–6.57 in the curdle at the extraction. In all CC cheeses, the pH ranged between 5.36 and 5.79 after 24 h of incubation.

During both BB and CC cheese processing, there was no significant difference (*p* > 0.05) until the curdle extraction. The pH of both BB and CC curdles from conventional farming, was significantly lower than respective CTRL cheeses. Moreover, after 24 h of ripening, the pH values of both BB and CC cheeses from the organic farm were significantly lower than the pH values of the cheeses enriched with BB and CC from the conventional farm.

Comparing BB and CC cheese processing, even if the pH of milk was significantly higher in BB than in CC batches (*p* < 0.05), after 24 h of incubation, BB cheese samples showed a significant decrease in pH to CC cheese samples (*p* < 0.0005).

### Microbial plate counts

**Figure 2:**
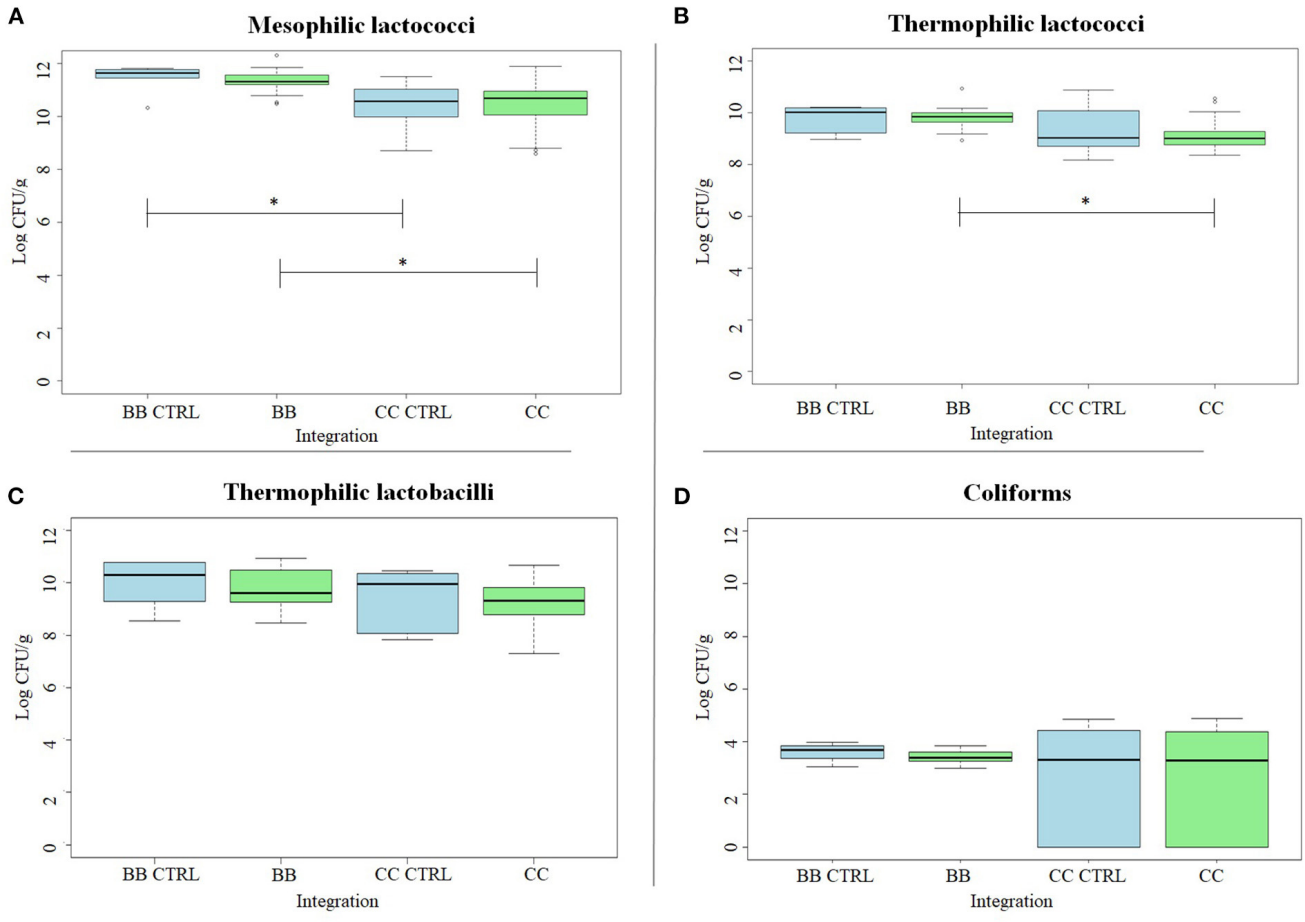
Box plots of observed mesophilic cocci **(A)**, thermophilic cocci **(B)**, thermophilic lactobacilli **(C)**, and coliforms **(D)**. Counts (Log CFU/g) in cheeses enriched with *Ribes nigrum* (BB) and *Cornus mas* (CC) and in respective CTRL (BB-CTRL and CC-CTRL) after 4 weeks of ripening. CTRL is colored in blue; enriched cheeses are colored in green. The thick horizontal black bars indicate the medians; the boxes indicate the range between the first (25th percentile) and the third quartile (75th percentile). The whiskers represent extreme values. Significant differences among CTRL and enriched cheeses are indicated by an asterisk (*: *p* < 0.05).

The Log CFU/g for each type of cheese and each tested medium are recorded in Figure 2 and Table 2.

**Table 2:** Microbial counts and Total Polyphenol Content (TPC) in cheeses after 4 weeks of ripening.

	**Amount (%)**	**Farming system**	**Mesophilic lactococci**	**Thermophilic lactococci**	**Thermophilic lactobacilli**	**Coliforms**	**TPC**
**Blackcurrant berries**							
CTRL-BB	0	-	11.4 ± 0.56^A^	9.8 ± 0.53^A^	10.0 ±0.92^A^	3.6 ± 0.34^A^	2.5 ± 0.88^A^
FD-BB	0.3	Organic	11.5 ± 0.20^A^	9.8 ± 0.24^A^	10.1 ± 0.24^A^	3.5 ± 0.13^A^	3.2 ± 1.17^AB^
FD-BB	0.6	Organic	11.3 ± 0.46^A^	9.7 ± 0.21^A^	9.5 ± 0.21^A^	3.5 ± 0.43^A^	3.0 ± 1.26^AB^
NFD-BB	0.3	Organic	11.3 ± 0.90^A^	9.8 ± 0.18^A^	9.7 ± 0.59^A^	3.4 ± 0.32^A^	2.4 ± 0.88^A^
NFD-BB	0.6	Organic	11.4 ± 0.19^A^	9.9 ± 0.25^A^	10.1 ± 0.79^A^	3.5 ± 0.20^A^	2.7 ± 1.03^AB^
FD-BB	0.3	Conventional	11.2 ± 0.69^A^	9.9 ± 1.0^A^	9.5 ± 1.3^A^	3.4 ± 0.32^A^	3.0 ± 0.78^AB^
FD-BB	0.6	Conventional	11.3 ± 0.07^A^	9.6 ± 0.38^A^	9.9 ± 0.49^A^	3.3 ± 0.07^A^	3.2 ± 0.91^B^
**Cornelian cherry**							
CTRL-CC	0	-	10.4 ± 1.0^A^	9.3 ± 0.99^AB^	9.4 ± 1.1^AB^	2.6 ± 2.1^A^	2.7 ± 0.71^A^
FD-CC	0.3	Organic	10.3 ± 1.12^A^	9.0 ± 0.58^AB^	9.5 ± 0.59^AB^	1.6 ±1.8^B^	2.9 ± 0.83^AB^
FD-CC	0.6	Organic	10.1 ± 1.25^A^	9.0 ± 0.03^AB^	9.4 ± 0.28^AB^	2.4 ± 2.4^AB^	2.9 ± 1.04^AB^
NFD-CC	0.3	Organic	11.1 ± 0.75^A^	9.5 ± 0.92^AB^	9.8 ± 0.59^A^	2.1 ± 1.7^AB^	2.8 ± 0.63^AB^
NFD-CC	0.6	Organic	11.2 ± 1.32^A^	9.9 ± 0.68^A^	10.2 ± 0.37^A^	1.4 ± 1.4^B^	3.1 ± 0.59^AB^
FD-CC	0.3	Conventional	11.0 ± 1.2^A^	9.4 ± 0.19^AB^	9.7 ± 0.80^AB^	3.2 ± 1.2^A^	3.2 ± 0.53^AB^
FD-CC	0.6	Conventional	10.0 ± 1.0^A^	8.8 ± 0.24^B^	8.7 ± 0.54^B^	3.5 ± 0.2^A^	3.5 ± 0.94^B^
NFD-CC	0.3	Conventional	10.1 ± 1.2^A^	8.9 ± 0.35^B^	8.6 ± 0.61^B^	3.0 ± 1.6^A^	3.2 ± 0.40^AB^
NFD-CC	0.6	conventional	10.5 ± 1.4^A^	9.3 ± 0.6^AB^	8.8 ± 0.43^B^	3.3 ± 1.4^A^	3.6 ± 0.59^B^

For each column and each enrichment (blackcurrant and cornelian cherry), bacterial count and TPC values with A and B superscripts are significantly different (p < 0.05, one-way ANOVA with post- hoc Tukey HSD).

Microbial counts are expressed as the mean value of Log CFU/g cheese ± standard deviation onto M17 at 30°C (mesophilic lactococci), M17 at 45°C (thermophilic lactococci), MRS at 45°C (thermophilic lactobacilli), and VRBA at 37°C (Coliforms). TPC is expressed as mg GAE equivalent per g of cheese. FD, freeze-dried; NFD, not freeze-dried; CTRL, Control; BB, blackcurrant berry; CC, cornelian cherry.

Thermophilic lactococci and lactobacilli mean values were about one order of magnitude lower than mesophilic lactococci. Coliforms' mean values were at least six orders of magnitude lower than LAB and very different comparing BB and CC cheeses: in BB cheeses, coliforms were in the range of 3.3–3.6 Log CFU/g and CC cheeses, they were in the range of 1.4–3.5 Log CFU/g (Figure 2). LAB and coliforms counts were higher in cheeses enriched with BB than CC, and the same trend was observed in respective CTRL cheeses: the CTRL cheeses made by the milk batch used for BB cheeses showed higher bacterial counts than the CTRL cheeses made by milk batch used for CC cheeses (Figure 2). Therefore, the difference observed is probably deriving from the milk rather than the specific BB or CC addition.

The dose of enrichment (0.3 vs. 0.6%), ingredient processing (freeze-dried vs. not-freeze-dried), and type of farming (organic vs. conventional) were compared by analyzing BB and CC cheeses separately (Table 2). Plate counts of thermophilic lactococci and lactobacilli were significantly lower in cheeses enriched with conventional than organic CC. By contrast, coliforms were significantly lower in the cheeses enriched with organic than in conventional CC. BB cheeses did not show any difference for any of the considered microbial groups.

### Polyphenol content

**Figure 3:**
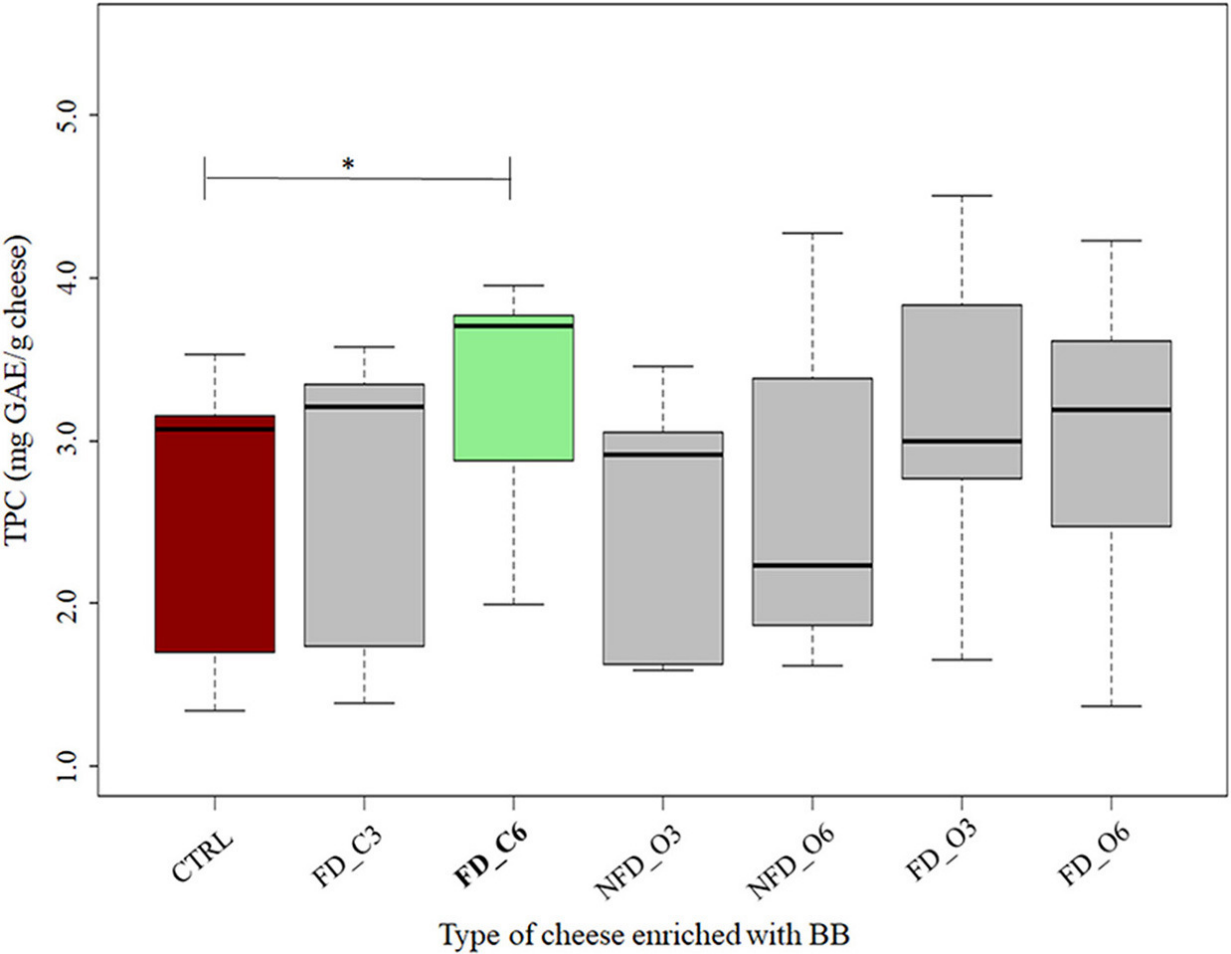
Total Polyphenol Content (TPC) in cheeses added with blackcurrant (BB) expressed in mg GAE/g cheese. CTRL: controls in red; NFD_C3: 0.3% not freeze-dried conventional BB cheeses; NFD_C6: 0.6% not freeze-dried conventional BB cheeses; FD_C3: 0.3% freeze-dried conventional BB cheeses; FD_C6: 0.6% freeze-dried conventional BB cheeses; FD_O3: 0.3% freeze-dried organic BB cheeses; FD_O6 0.6% freeze-dried organic BB cheeses; NFD_O3: 0.3% not freeze-dried organic BB cheeses; NFD_O6: 0.6% not freeze-dried organic BB cheeses. The thick horizontal black bars indicate the medians; the boxes indicate the range between the first (25th percentile) and the third quartile (75th percentile). The whiskers represent extreme values. Significant differences between CTRL and enriched cheeses are indicated by an asterisk (*: *p*-values < 0.05). *n* = 3 for enriched cheeses; *n* = 6 for CTRL cheeses. The TPC boxes significantly different are in green color.

**Figure 4:**
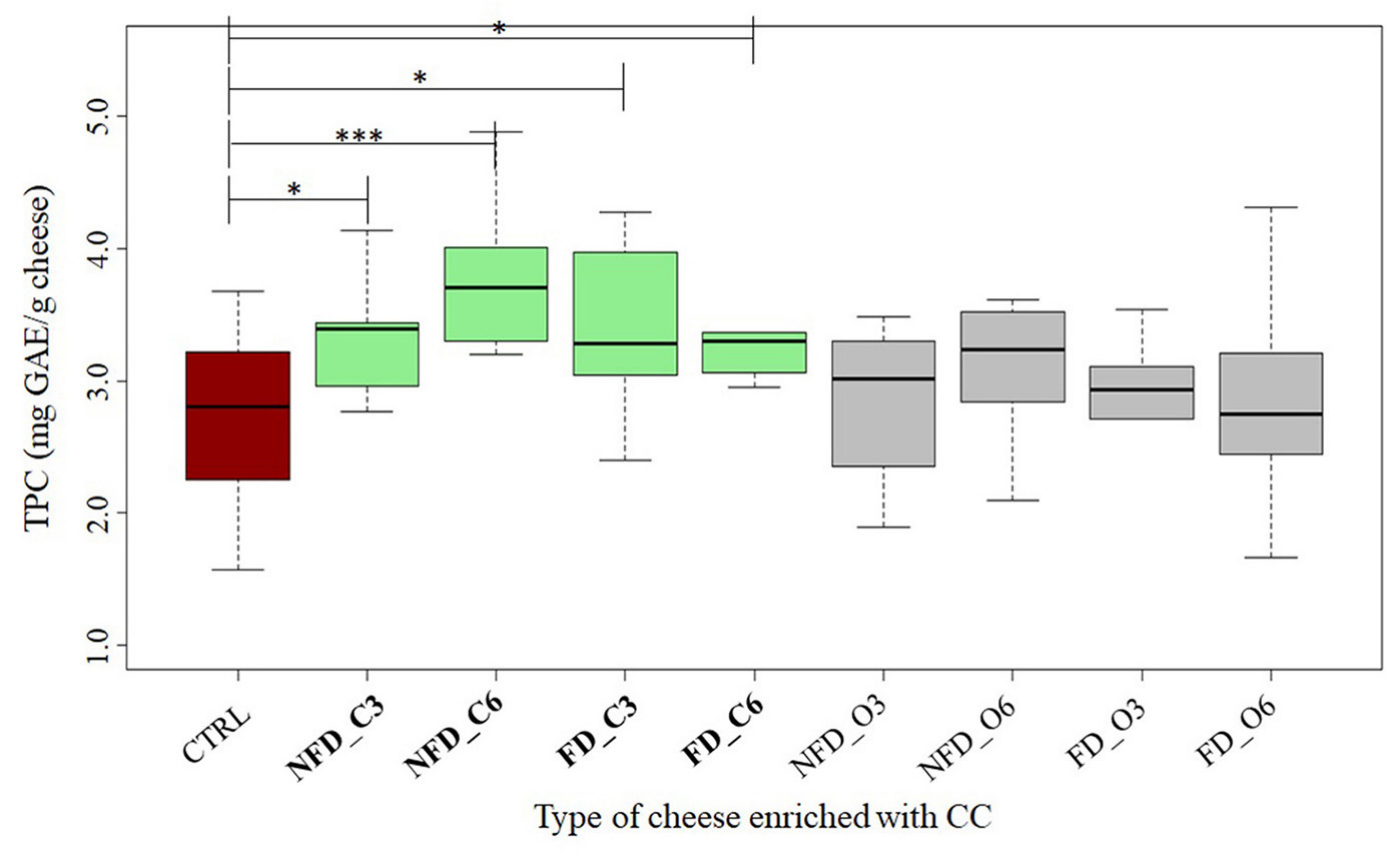
Total Polyphenol Content (TPC) in cheeses added with Cornelian cherry (CC) expressed in mg GAE/g cheese. CTRL: controls in red; NFD_C3: 0.3% not freeze-dried conventional CC cheeses; NFD_C6: 0.6% not freeze-dried conventional CC cheeses; FD_C3: 0.3% freeze-dried conventional CC cheeses; FD_C6: 0.6% freeze-dried conventional CC cheeses; FD_O3: 0.3% freeze-dried organic CC model cheeses; FD_O6 0.6% freeze-dried organic CC cheeses; NFD_O3: 0.3% not freeze-dried organic CC cheeses; NFD_O6: 0.6% not freeze-dried organic CC cheeses. The thick horizontal black bars indicate the medians; the boxes indicate the range between the first (25th percentile) and the third quartile (75th percentile). The whiskers represent extreme values. Significant differences between CTRL and enriched cheeses are indicated by an asterisk (*: *p* < 0.05; ***: *p* < 0.001). The number of replicates: *n* = 3 for all enriched model cheeses; *n* = 6 for CTRL. The TPC boxes significantly different are in green color.

Analyzing the TPC in the enrichment ingredient samples, we could see the mean values of 1,525 and 1,097 mg GAE per 100 g of raw blackcurrant and Cornelian cherry samples, respectively. The TPC values in cheeses added with BB (Figure 3) and CC (Figure 4) are listed in Table 2. All the conventional BB cheeses had higher TPC than CTRL cheeses and the difference was significant when 0.6% of conventional BB was added (3.2 mg GAE/g cheese). The organic BB increased TPC in cheeses from 2.5 in CTRL cheeses to a maximum value of 3.2 mg GAE/g when freeze-dried organic BB was added at 0.6%. Cheeses enriched with CC showed higher TPC than BB cheeses and CTRL cheeses (2.7 mg GAE/g cheese). Cornelian cherries from conventional farming were able to significantly increase the cheese TPC to a maximum value of 3.6 mg GAE/g cheese when not freeze-dried conventional CC was used; while with the organic CC, a significantly higher TPC was observed when not freeze-dried CC was used at 0.6% (2.9 mg GAE/g cheese).

### NMR metabolite profile of cheeses

The quantitative evaluation of the aqueous and lipidic extracts of the Caciotta-like cheeses is shown in Supplementary Table 1. The NMR spectrum of the aqueous extracts exhibited sharp peaks, assigned to free amino acids (FAAs), organic acids such as acetic, citric, formic, pyruvic, lactic, and tartaric acids, and other metabolites (glucose, 2,3-butanediol, ethanol, DMS, histamine, and lactose). No significant difference was reported for 0.3% enriched cheeses. To highlight differences due to the 0.6% dose of blackcurrant or Cornelian cherry, the metabolite levels in 0.6% enriched and CTRL cheeses are compared, as reported in Table 3. Aspartic acid, lactic acid, GABA, and histamine significantly increased when cheeses were enriched with 0.6% BB, with histamine displaying a higher accumulation in 0.6% enriched BB cheeses than in CTRL and 0.6% CC cheeses (FC of 1.45 and 1.83 compared with CTRL and CC cheeses, respectively). Alpha-D-glucose and beta-D-glucose showed a significantly lower accumulation in 0.6% BB cheeses, whereas no significant changes were detected in acetic acid, 2,3-butanediol, citric acid, DMS, ethanol, lactose, leucine, and tartaric acid (Table 3).

**Table 3:** Identified compounds in CTRL and cheeses enriched with 0.6% of BB and CC by NMR analysis.

**Compounds**	**BB vs. CTRL-BB cheeses**	**BB vs. CC cheeses**	**CC vs. CTRL-CC cheeses**
**Acids**			
Acetic acid	−1.01	−1.01	1.00
Citric acid	1.06	−1.00	1.07
Formic acid	−1.02	1.56^*^	−1.59
Pyruvic acid	1.41	−1.43	2.03^***^
Lactic acid	1.13^*^	1.52^***^	−1.35^***^
Tartaric acid	−1.16	−1.2	1.04
**Sugars**			
Alpha-D-glucose	−1.47^*^	−1.71^***^	1.17
Beta-D-glucose	−1.33^*^	−1.53^**^	1.15
Lactose	1.09	1.01	1.08
Arginine	1.14	1.10	1.04
**Amino acids**			
Aspartic acid	1.17^*^	1.26^***^	−1.08
Leucine	1.08	−1.14	1.22
GABA^B^	1.14^*^	1.26^***^	−1.11
Tyrosine	1.15	1.12	1.03
**Others**			
2,3-Butanediol	1.10	−1.03	1.13
Ethanol	1.10	1.08	1.01
DMS^A^	1.14	1.22	−1.06
Histamine	1.45^**^	1.83^***^	−1.27*

Significance ^*^p < 0.05, ^**^p < 0.001, ^***^p < 0.0001.

^A^ DMS: Dimethylsulfide.

^B^ GABA: γ-Aminobutyric acid.

Fold changes (FC) of compound accumulation in 0.6% BB, 0.6% CC, and CTRL cheeses have been considered. Positive FC indicates an increase in metabolite concentration; negative FC indicates a decrease in metabolite concentration.

There was no significant difference in the metabolite profile comparing the supplier farm (organic or conventional, Supplementary Table 1) or the ingredient processing (freeze-drying or not freeze-drying) indicating that only the ingredient (blackcurrant or Cornelian cherry) and the dose (0.3% or 0.6%) are the only discriminants in the final definition of the cheese composition.

**Figure 5:**
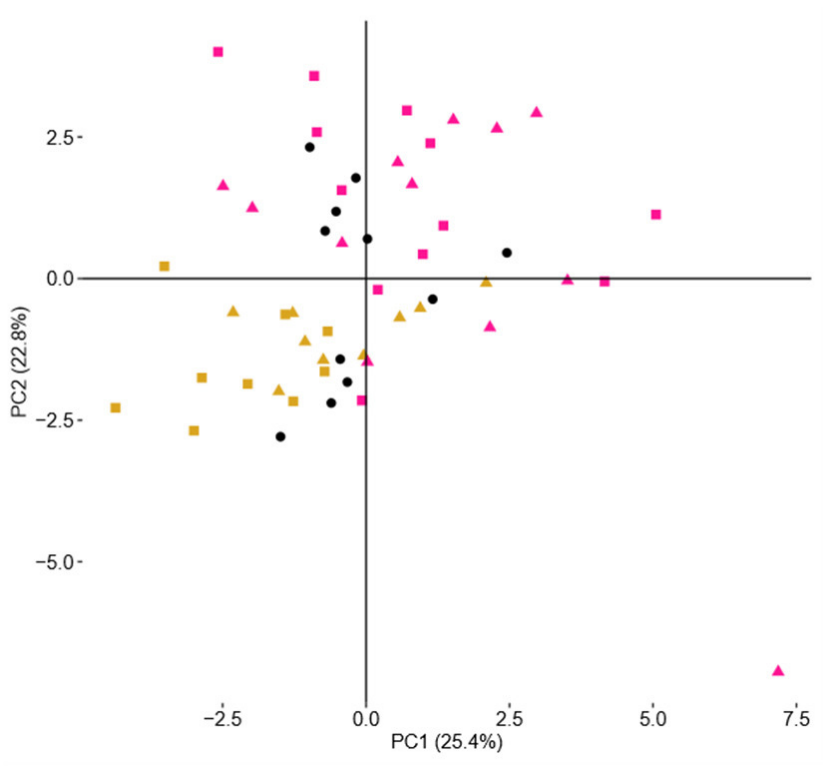
Scores plot of a two-component PCoA model of NMR spectra showing sample clustering according to ingredient addition: CTRL cheeses (black circles), BB cheeses (yellow), and CC cheeses (pink). Squares are for the addition of 0.6% and triangles for the addition of 0.3% of the ingredient.

**Figure 6:**
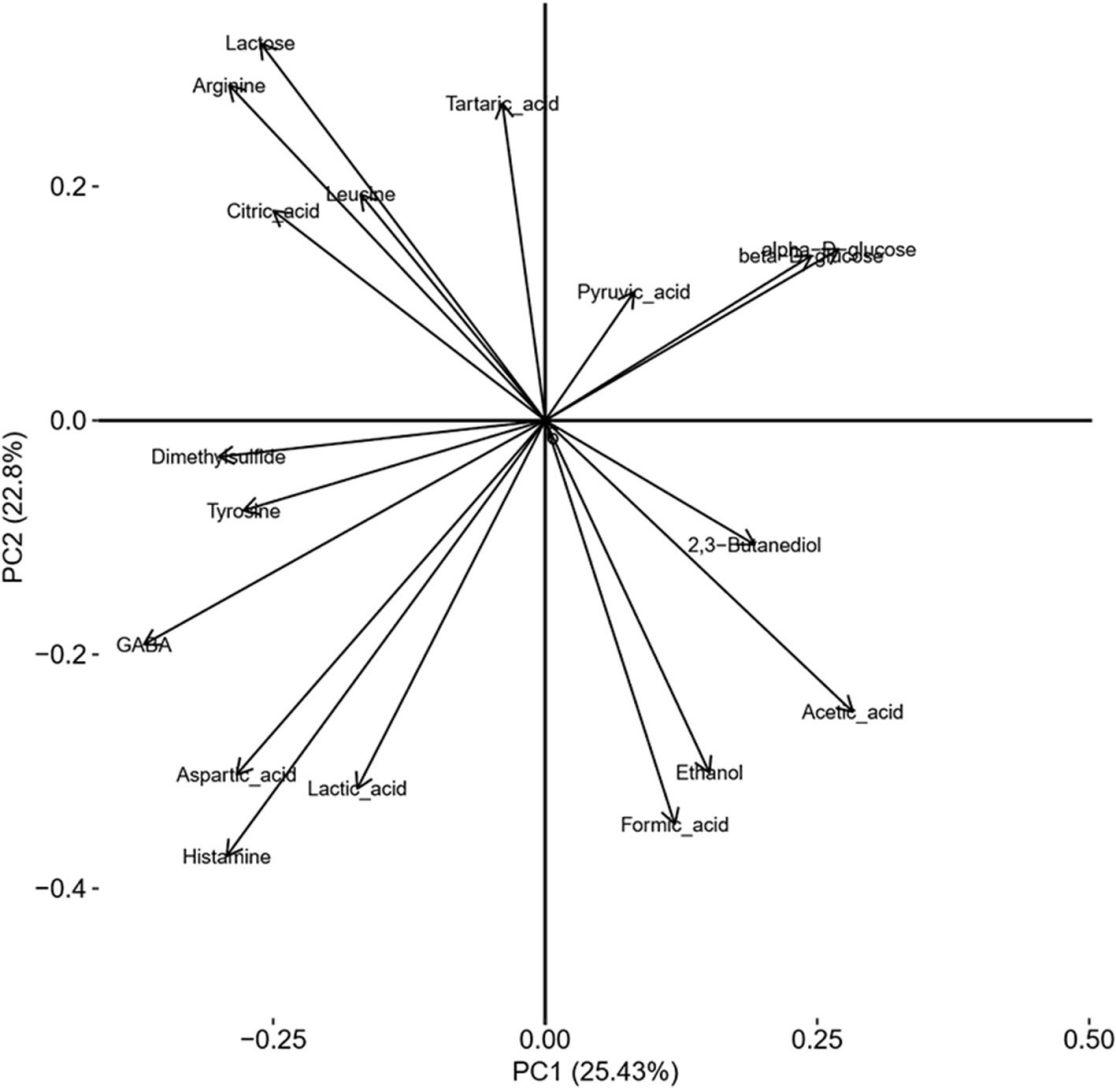
Loading plot of a two-component PCoA model of NMR spectra showing sample clustering according to ingredient addition.

PCoA of the NMR spectra from aqueous extracts of the cheese samples for the differences in terms of added ingredients (BB or CC) is presented in Figure 5. The representative points of the cheese samples are mapped in the space spanned by the first two principal components: PC1 vs. PC2. This scores plot is illustrating a reasonable clustering appearing according to the enrichment with 25% of variance captured by the first PC and 23% by the second PC. To find out precisely which regions of the NMR spectra have caused the separation among the CTRL, BB, and CC cheeses, the loading plot of the related PCoA model is shown in Figure 6. This loading plot shows the regions of the NMR spectra, which are responsible for the clustering appearing in the scores plot of the cheeses. Peaks with different levels between the BB and CC enrichments appeared in the same region of the scores (Figure 3) and the loadings plot (Figure 6). In Figure 5, it is clearly shown that PC2 is capturing most of the variations between the BB and CC enrichments. The inspection of Figure 4 indicates that GABA, histamine, and organic acids such as lactic acid, formic acid, and aspartic acid levels are found somewhat higher in BB than in CTRL and CC cheeses, and by contrast, lower levels of glucose monosaccharides: alpha-D-glucose and beta-D-glucose are found in BB than in CTRL and CC cheeses. The scrutiny of the higher-order PCs (three, four, and five PCs) indicated no additional separation between enrichment.

Regarding the NMR spectrum of the lipidic extracts, no significant difference was noticed comparing CTRL, BB, and CC cheeses (Supplementary Table 1).

### Organoleptic evaluation

The relationships between enrichment with BB or CC and organoleptic characteristics of cheeses are summarized in Table 4. There was no significant difference (*p* > 0.05) in the odor, texture, and taste characteristics of cheeses. The panelists gave a significantly higher score to the color of CTRL than BB cheeses.

**Table 4:** Organoleptic evaluation of processed cheeses after 4 weeks of ripening, as affected by enrichment (blackcurrant or Cornelian cherry) dose of the enrichment (0.3 or 0.6%) and farming system (conventional or organic).

	**Amount (%)**	**Farming system**	**Color**	**Odor**	**Taste**	**Texture**	**Total score**
**Blackcurrant berries**							
CTRL-BB	0	-	7.8 ± 0.6^A^	7.0 ± 1.0^A^	6.8 ± 1.1^A^	6.9 ± 1.1^A^	28.5 ± 2.5^A^
FD-BB	0.3	Organic	6.8 ± 0.9^B^	7.1 ± 1.1^A^	7.2 ± 1.0^A^	6.8 ± 1.2^A^	27.9 ± 3.1^A^
FD-BB	0.6	Organic	6.4 ± 1.0^C^	7.1 ± 1.2^A^	7.1 ± 1.4^A^	7.1 ± 1.0^A^	27.7 ± 3.3^A^
NFD-BB	0.3	Organic	6.8 ± 0.8^B^	7.1 ± 1.0^A^	6.8 ± 1.2^A^	6.7 ± 0.9^A^	27.4 ± 2.6^A^
NFD-BB	0.6	Organic	6.7 ± 1.0^B^	6.7 ± 1.2^A^	6.8 ± 1.1^A^	6.7 ± 0.8^A^	26.8 ± 3.1^A^
FD-BB	0.3	Conventional	6.8 ± 0.8^B^	7.0 ± 1.1^A^	6.7 ± 1.1^A^	7.1 ± 0.9^A^	27.7 ± 2.9^A^
FD-BB	0.6	Conventional	6.8 ± 1.1^B^	7.0 ± 1.1^A^	6.7 ± 1.6^A^	6.6 ± 1.1^A^	27.1 ± 3.8^A^
**Cornelian cherry**							
CTRL-CC	0	-	7.7 ± 0.9^A^	7.4 ± 0.7^A^	7.2 ± 0.9^A^	7.1 ± 0.9^A^	29.5 ± 2.5^A^
FD-CC	0.3	Organic	7.0 ± 0.9^AB^	6.9 ± 0.9^A^	7.1 ± 0.9^A^	6.7 ± 1.0^A^	27.7 ± 2.2^A^
FD-CC	0.6	Organic	6.9 ± 0.9^AB^	7.3 ± 0.7^A^	6.7 ± 1.1^A^	6.6 ± 0.9^A^	27.5 ± 2.4^A^
NFD-CC	0.3	Organic	7.4 ± 0.8^AB^	7.1 ± 0.9^A^	7.1 ± 1.3^A^	6.9 ± 1.1^A^	28.6 ± 3.3^A^
NFD-CC	0.6	Organic	6.7 ± 1.1^AB^	7.6 ± 1.1^A^	6.7 ± 1.1^A^	7.0 ± 0.8^A^	28.0 ± 3.3^A^
FD-CC	0.3	Conventional	7.2 ± 1.0^AB^	7.5 ± 0.9^A^	6.8 ± 1.0^A^	6.9 ± 1.0^A^	28.3 ± 3.1^A^
FD-CC	0.6	Conventional	6.9 ± 0.8^AB^	7.4 ± 0.8^A^	6.9 ± 1.0^A^	6.7 ± 1.0^A^	27.8 ± 2.5^A^
NFD-CC	0.3	Conventional	6.9 ± 0.8^AB^	7.4 ± 0.7^A^	7.3 ± 1.2^A^	6.8 ± 1.0^A^	28.4 ± 2.4^A^
NFD-CC	0.6	Conventional	6.6 ± 0.9^B^	7.3 ± 0.9^A^	6.8 ± 1.0^A^	6.7 ± 1.1^A^	27.3 ± 2.9^A^

For each column and each enrichment (blackcurrant and cornelian cherry), organoleptic scores values with A and B superscripts are significantly different (p < 0.05, one-way ANOVA with post-hoc Tukey HSD).

FD, freeze-dried; NFD, not freeze-dried; CTRL, Control; BB, blackcurrant berry; CC, cornelian cherry. The scores are represented as mean values ± standard deviation. The Total Score is the sum of all four scores of the four attributes.

## Discussion

The aim of this study was to evaluate the effects of the BB and CC enrichment in Caciotta-like cheeses not only on TPC but also on the microbial population, the organoleptic attributes, and the composition of the cheeses at the end of the ripening.

In general, the addition of BB and CC to cheese did not have a severe impact on cheese production.

The acidification process was monitored during the steps of cheese manufacturing. The results of the analysis showed a decrease in pH in all the cheeses, according to expectations. The starter cultures worked efficiently without inhibition neither by BB nor CC enrichment, and acidification activity was always lowering the pH in the cheese.

Considering microbial counts, the addition of BB in cheese did not significantly change the counts of LAB or coliforms. By converse, the addition of CC and in particular conventional CC was affecting the counts of thermophilic LAB (lactococci and lactobacilli) and coliforms, and in particular, when the thermophilic LAB was significantly lower, the coliforms were significantly higher. In the literature, the antimicrobial activity of extracts of Cornelian cherry fruits is generally known (63, 64), but to the best of our knowledge, there is no study about the differences in antibacterial activities between conventional and organic Cornelian cherries.

About the TPC evaluation, blackcurrant and Cornelian cherry showed values of 1.53 and 1.10 mg GAE/100 g of berries, respectively. Blackcurrant TPC is in agreement with previous works (65); Cornelian cherry TPC is 10-fold higher than the values found in other studies (66). Dzydzan et al. (67) showed that the high variability of the TPC in Cornelian cherries is related to the cultivar and fruit color. Considering the TPC measured in cheese, our analysis showed that even if TPC values were higher in blackcurrant than in Cornelian cherries, by converse, BB cheeses showed lower TPC than CC cheeses.

Comparing BB- and CC-enriched cheeses with their respective CTRL, higher TPC was found only when BB and CC from conventional farming were used and in particular at 0.6% dose, whereas no significant difference was found between cheeses enriched with BB and CC from organic farming. Freeze-drying of BB and CC was not a key factor in the TPC of enriched cheeses. We speculated that the typology of farming (organic or conventional) altered the profile of the produced polyphenols. The differences in the polyphenol profiles are not indicated by the Folin–Ciocalteu test, which is not a specific method for polyphenols, since it is based on the reductive potential of all the compounds which may react with the reagent (55). The polyphenols derived from conventional farming, even if in similar amounts, could be different compared with the ones produced under organic farming. This explanation could also fit with the microbial plate counts: the different profiles of polyphenol compounds from conventional farming could have different and selective antimicrobial activities that were more evident when conventional CC was used as enrichment in cheeses. To the best of our knowledge, there is no work with information about the effect of different types of polyphenols on different microbial groups. Furthermore, model cheeses are in need to be manufactured with higher amounts of blackcurrant and Cornelian cherry to check this speculation, and accurate profiling of polyphenols in BB and CC is in progress to understand if the observed antibacterial activity against thermophilic LAB is depending on the type of polyphenol compounds.

Regarding the metabolite composition of the cheeses, we found that acetic acid, 2,3-butanediol, dimethyl sulfide, ethanol, leucine, and pyruvic and tartaric acids were not impacted by the enrichment, probably because these compounds are found as common neither in cheese nor in blackcurrant and Cornelian cherry (37, 38). Several compounds with different concentrations depending on the blackcurrant and Cornelian cherry cheese enrichment were found. The significantly higher amount of lactate and significantly lower amount of lactose and glucose (constituent monosaccharide of lactose) in 0.6% BB-enriched cheeses than in CTRL and CC cheeses could be indicative of higher lactose metabolism, and therefore of the impact that BB could have on the LAB which are the effective main factors in the production of the lactate from lactose (68). LAB metabolized lactose by fermentative pathways, producing also other compounds such as formic acid. The fermentation of the sugars in organic acids is an essential event in cheese manufacturing for the production of good quality cheese, since the presence of left fermentable carbohydrates may lead to the development of undesirable secondary flora. In parallel with the sugar decrease, in 0.6% BB cheeses, we could see a significant increase in organic acids and, in particular, lactic acid which is the major product of carbohydrate catabolism by LAB (68). Histamine is a biogenic amine that again was found significantly higher in BB than CC cheeses and could be a LAB product from the decarboxylation of histidine in cheese (69). Histamine could have toxicological effects. The provisions of European Union regulations specify a permissible level of histamine only for fish and fish products, (70) and there are no established criteria relating to the level of this amine in cheeses. The US Food and Drug Administration considers a danger to health if the histamine level is higher than 500 mg/ Kg (71), and our cheeses always showed histamine amounts lower than this cutoff.

Cheeses enriched with 0.6% BB also showed increased significant levels of several amino acids, including arginine, aspartic acid, GABA, and tyrosine that again were consistent with the idea that BB could positively impact the metabolism of the caseins in free amino acids, the consequence of a successful LAB proteolysis activity, which is the major process in the formation of the texture, flavor, and aroma during cheese ripening (68). In particular, regarding GABA production, it was one of the main distinctive compounds produced in BB cheeses. GABA is usually produced in cheese by glutamic acid decarboxylation by LAB (72), and this compound could be a further confirmation of the positive effect of blackcurrant on the LAB activities. The previous study showed that some lactobacilli species, also used as a dairy starter, could be positively affected in their growth by phenolic extracts such as catechins and epicatechins (73), and are very common in blackcurrant. GABA is a bioactive compound with the potential for beneficial effects in humans (72, 74), and the GABA increase following the addition of BB to cheeses has to be further investigated through *in vitro* tests.

We speculated that these significant differences in metabolic profile between BB vs. CC and CTRL cheeses could be caused by some positive modulation of blackcurrant on LAB. This positive modulation could be attributable to an attitude of the LABs more ready to metabolize the polyphenols typical of blackcurrant than those of Cornelian cherry. The LAB polyphenols metabolism could be a compound selective toward polyphenols present in blackcurrant than the ones present in Cornelian cherry. From the literature, polyphenol profiles of blackcurrant and Cornelian cherry are very different: blackcurrant is rich in delphinidin 3-O-rutinoside and cyanidin-3-O-rutinoside while two of the main compounds present in Cornelian cherries are cyaniding-3-O-galactoside and pelargonidin-3-O-galactoside (47, 75). The use of blackcurrant polyphenols by dairy LAB could explain the fact that although blackcurrant had higher polyphenol content, the respective cheeses showed a lower TPC than those enriched with Cornelian cherries.

## Conclusion

In conclusion, our results show that the cheese enrichment with conventional blackcurrant and Cornelian cherry was able to improve TPC, by increasing the bioactive potential of these cheeses, without altering the taste and the overall organoleptic evaluation. We never noticed the undesired effect of polyphenols on the LAB community, confirming the possibility of the use of both blackcurrant and Cornelian cherry in cheeses even at the higher tested concentration (0.6%). Analyzing the NMR results, it is evident that a metabolite compositional change in 0.6% BB cheeses could be indicative of an active bacterial fermentation process ascribed to the activity of the LAB added as a starter. Although different factors may influence the cohabitation of LAB in the cheeses, the results can be read in terms of the positive effect of blackcurrant on the growth of LAB in the cheese. Additional studies *in vitro* could be very useful to test the activity of blackcurrant extracts on LAB. A possible next step would be to scale up the production of the enriched Caciotta-like cheese in a cheese factory and to evaluate the shelf life of this product.

## Data availability statement

The original contributions presented in the study are included in the article/Supplementary material, further inquiries can be directed to the corresponding author.

## Author contributions

EF conceived and designed the study and wrote the paper. JA and MB analyzed the data and wrote the manuscript. JA, MB, AM, TN, and PS performed the experiments. LB, TN, and RL participated in the critical revision of the methods of the experiment. All authors read and approved the final manuscript.

## References

[1] Stiller A Garrison K Gurdyumov K Kenner J Yasmin F Yates P. From fighting critters to saving lives: polyphenols in plant defense and human health. Int J Mol Sci. (2021) 22:8995 10.3390/ijms22168995 34445697 PMC8396434

[2] Rezai-Zadeh K Shytle D Sun N Mori T Hou H Jeanniton D. Green tea epigallocatechin-3-gallate (EGCG) modulates amyloid precursor protein cleavage and reduces cerebral amyloidosis in Alzheimer transgenic mice. J Neurosci. (2005) 25:8807–14. 10.1523/JNEUROSCI.1521-05.2005 16177050 PMC6725500

[3] Lim GP Chu T Yang F Beech W Frautschy SA Cole GM. The curry spice curcumin reduces oxidative damage and amyloid pathology in an Alzheimer transgenic mouse. J Neurosci. (2001) 21:8370–7. 10.1523/JNEUROSCI.21-21-08370.2001 11606625 PMC6762797

[4] Satoh T Izumi M Inukai Y Tsutsumi Y Nakayama N Kosaka K. Carnosic acid protects neuronal HT22 cells through activation of the antioxidant-responsive element in free carboxylic acid- and catechol hydroxyl moieties-dependent manners. Neurosci Lett. (2008) 434:260–5. 10.1016/j.neulet.2008.01.079 18329808

[5] Larrosa M Luceri C Vivoli E Pagliuca C Lodovici M Moneti G. Polyphenol metabolites from colonic microbiota exert anti-inflammatory activity on different inflammation models. Mol Nutr Food Res. (2009) 53:1044–54. 10.1002/mnfr.200800446 19557820

[6] Radnai B Tucsek Z Bognar Z Antus C Mark L Berente Z. Ferulaldehyde, a water-soluble degradation product of polyphenols, inhibits the lipopolysaccharide-induced inflammatory response in mice. J Nutr. (2009) 139:291–7. 10.3945/jn.108.097386 19106314

[7] Kostyuk VA Potapovich AI Suhan TO De Luca C Korkina LG. Antioxidant and signal modulation properties of plant polyphenols in controlling va.scular inflammation. Eur J Pharmacol. (2011) 658:248–56. 10.1016/j.ejphar.2011.02.022 21371465

[8] González-Sarrías A Azorín-Ortuño M Yáñez-Gascón MJ Tomás-Barberán FA García-Conesa MT Espín JC. Dissimilar in vitro and in vivo effects of ellagic acid and its microbiota-derived metabolites, urolithins, on the cytochrome P450 1A1. J Agric Food Chem. (2009) 57:5623–32. 10.1021/jf900725e 19469472

[9] Seeram NP Aronson WJ Zhang Y Henning SM Moro A Lee RP. Pomegranate ellagitannin-derived metabolites inhibit prostate cancer growth and localize to the mouse prostate gland. J Agric Food Chem. (2007) 55:7732–7. 10.1021/jf071303g 17722872

[10] Greenberg JA. Chocolate intake and diabetes risk. Clin Nutr. (2015) 34:129–33. 10.1016/j.clnu.2014.02.005 24582922

[11] Crichton GE Elias MF Dearborn P Robbins M. Habitual chocolate intake and type 2 diabetes mellitus in the maine-syracuse longitudinal study: (1975–2010): prospective observations. Appetite. (2017) 108: 263–9. 10.1016/j.appet.2016.10.008 27725277

[12] Loke WM Proudfoot JM Stewart S McKinley AJ Needs PW Kroon PA. Metabolic transformation has a profound effect on anti-inflammatory activity of flavonoids such as quercetin: lack of association between antioxidant and lipoxygenase inhibitory activity. Biochem Pharmacol. (2008) 75:1045–53. 10.1016/j.bcp.2007.11.002 18096136

[13] Bondonno CP Yang X Croft KD Considine MJ Ward NC Rich L. Flavonoid-rich apples and nitrate-rich spinach augment nitric oxide status and improve endothelial function in healthy men and women: a randomized controlled trial. Free Radic Biol Med. (2012) 52:95–102. 10.1016/j.freeradbiomed.2011.09.028 22019438

[14] Schroeter H Heiss C Balzer J Kleinbongard P Keen CL Hollenberg NK. (-)-Epicatechin mediates beneficial effects of flavanol-rich cocoa on vascular function in humans. Proc Natl Acad Sci U S A. (2006) 103:1024–9. 10.1073/pnas.0510168103 16418281 PMC1327732

[15] Heiss C Finis D Kleinbongard P Hoffmann A Rassaf T Kelm M. Sustained increase in flow-mediated dilation after daily intake of high-flavanol cocoa drink over 1 week. J Cardiovasc Pharmacol. (2007) 49:74–80. 10.1097/FJC.0b013e31802d0001 17312446

[16] Shen Y Croft KD Hodgson JM Kyle R Lee ILE Wang Y. Quercetin and its metabolites improve vessel function by inducing eNOS activity via phosphorylation of AMPK. Biochem Pharmacol. (2012) 84:1036–44. 10.1016/j.bcp.2012.07.016 22846602

[17] Lee HC Jenner AM Low CS Lee YK. Effect of tea phenolics and their aromatic fecal bacterial metabolites on intestinal microbiota. Res Microbiol. (2006) 157:876–84. 10.1016/j.resmic.2006.07.004 16962743

[18] Tzounis X Rodriguez-Mateos A Vulevic J Gibson GR Kwik-Uribe C Spencer JPE. Prebiotic evaluation of cocoa-derived flavanols in healthy humans by using a randomized, controlled, double-blind, crossover intervention study. Am J Clin Nutr. (2011) 93:62–72. 10.3945/ajcn.110.000075 21068351

[19] Tzounis X Vulevic J Kuhnle GGC George T Leonczak J Gibso GR. Flavanol monomer-induced changes to the human faecal microflora. Br J Nutr. (2008) 99:782–92. 10.1017/S0007114507853384 17977475

[20] Wiswedel I Hirsch D Kropf S Gruening M Pfister E Schewe T. Flavanol-rich cocoa drink lowers plasma F2-isoprostane concentrations in humans. Free Radic Biol Med. (2004) 37:411–21. 10.1016/j.freeradbiomed.2004.05.013 15223075

[21] Caccetta RA-A Burke V Mori TA Beilin LJ Puddey IB Croft KD. Red wine polyphenols, in the absence of alcohol, reduce lipid peroxidative stress in smoking subjects. Free Radic Biol Med. (2001) 30:636–42. 10.1016/S0891-5849(00)00497-4 11295361

[22] Loke WM Hodgson JM Proudfoot JM McKinley AJ Puddey IB Croft KD. Pure dietary flavonoids quercetin and (-)-epicatechin augment nitric oxide products and reduce endothelin-1 acutely in healthy men. Am J Clin Nutr. (2008) 88:1018–25. 10.1093/ajcn/88.4.1018 18842789

[23] Olaleye MT Rocha BTJ. Acetaminophen-induced liver damage in mice: effects of some medicinal plants on the oxidative defense system. Exp Toxicol Pathol. (2008) 59:319–27. 10.1016/j.etp.2007.10.003 18054472

[24] Nicholson SK Tucker GA Brameld JM. Physiological concentrations of dietary polyphenols regulate vascular endothelial cell expression of genes important in cardiovascular health. Br J Nutr. (2010) 103:1398–403. 10.1017/S0007114509993485 20021702

[25] Han YS Zheng WH Bastianetto S Chabot JG Quirion R. Neuroprotective effects of resveratrol against β-amyloid-induced neurotoxicity in rat hippocampal neurons: involvement of protein kinase C. Br J Pharmacol. (2004) 141:997–1005. 10.1038/sj.bjp.0705688 15028639 PMC1574264

[26] Dasgupta B Milbrandt J. Resveratrol stimulates AMP kinase activity in neurons. Proc Natl Acad Sci U S A. (2007) 104:7217–22. 10.1073/pnas.0610068104 17438283 PMC1855377

[27] Shen Y Ward NC Hodgson JM Puddey IB Wang Y Zhang D. Dietary quercetin attenuates oxidant-induced endothelial dysfunction and atherosclerosis in apolipoprotein e knockout mice fed a high-fat diet: a critical role for heme oxygenase-1. Free Radic Biol Med. (2013) 65:908–15. 10.1016/j.freeradbiomed.2013.08.185 24017971

[28] Darnton-Hill I Mora JO Weinstein H Wilbur S Nalubola PR. Iron and folate fortification in the Americas to prevent and control micronutrient malnutrition: an analysis. Nutr Rev. (1999) 57:25–31. 10.1111/j.1753-4887.1999.tb01773.x 10047703

[29] Sanchez-Bel P Romojaro A Egea I Pretel MT. Wild edible plants as potential antioxidant or nutritional supplements for beverages minimally processed. LWT. (2015) 62:830–7. 10.1016/j.lwt.2014.06.017

[30] Apostolidis E Kwon YI Shetty K. Inhibitory potential of herb, fruit, and fungal-enriched cheese against key enzymes linked to type 2 diabetes and hypertension. Innov Food Sci Emerg Technol. (2007) 8:46–54. 10.1016/j.ifset.2006.06.001

[31] Jaster H Arend GD Rezzadori K Chaves VC Reginatto FH Petrus JCC. Enhancement of antioxidant activity and physicochemical properties of yogurt enriched with concentrated strawberry pulp obtained by block freeze concentration. Food Res Int. (2018) 104:119–25. 10.1016/j.foodres.2017.10.006 29433776

[32] Lucera A Costa C Marinelli V Saccotelli MA Del Nobile MA Conte A. Fruit and vegetable by-products to fortify spreadable cheese. Antioxidants. (2018) 7:61. 10.3390/antiox7050061 29693632 PMC5981247

[33] Carafa I Stocco G Nardin T Larcher R Bittante G Franciosi E. Production of naturally γ-aminobutyric acid-enriched cheese using the dairy strains *Streptococcus thermophilus* 84C and *Lactobacillus brevis* DSM 32386. Front Microbiol. (2019) 10:1–11. 10.3389/fmicb.2019.00093 30814980 PMC6381070

[34] Gobbetti M Neviani E Fox P Varanini G.M. The Cheeses of Italy: Science and technology. New York, NY: Springer International Publishing (2018).

[35] Bimbo F Bonanno A Nocella G Viscecchia R Nardone G De Devitiis B. Consumers' acceptance and preferences for nutrition-modified and functional dairy products: a systematic review. Appetite. (2017) 113:141–54. 10.1016/j.appet.2017.02.031 28235616

[36] Granato D Santos JS Salem RDS Mortazavian AM Rocha RS Cruz AG. Effects of herbal extracts on quality traits of yogurts, cheeses, fermented milks, and ice creams: a technological perspective. Curr Opinion Food Sci. (2018) 19:1–7. 10.1016/j.cofs.2017.11.013

[37] Cyboran S Bonarska-Kujawa D Pruchnik H Zyłka R Oszmiański J Kleszczyńska H. Phenolic content and biological activity of extracts of blackcurrant fruit and leaves. Food Res Int. (2014) 65:47–58. 10.1016/j.foodres.2014.05.03733547351

[38] Bayram HM Ozturkcan AS. Bioactive components and biological properties of cornelian cherry (*Cornus mas* L.): a comprehensive review. J Funct Foods. (2020) 75:104252. 10.1016/j.jff.2020.104252

[39] De Biaggi M Donno D Mellano MG Riondato I Rakotoniaina EN Beccaro GL. *Cornus mas* (L.) fruit as a potential source of natural health-promoting compounds: physico-chemical characterisation of bioactive components. Plant Foods Hum Nutr. (2018) 73:89–94. 10.1007/s11130-018-0663-4 29671173

[40] Sozański T Kucharska AZ Rapak A Szumny D Trocha M Merwid-Lad A. Iridoid–loganic acid versus anthocyanins from the Cornus mas fruits (cornelian cherry): common and different effects on diet-induced atherosclerosis, PPARs expression and inflammation. Atherosclerosis. (2016) 254:151–60. 10.1016/j.atherosclerosis.2016.10.001 27744131

[41] Raikos V Ni H Hayes H Ranawana V. Antioxidant properties of a yogurt beverage enriched with salal (*Gaultheria shallon*) berries and blackcurrant (*ribes nigrum*) pomace during cold storage. Beverages. (2019) 5:1–11. 10.3390/beverages5010002

[42] Topdaş EF Çakmakçi S Çakiroglu K. The antioxidant activity, vitamin C contents, physical, chemical and sensory properties of ice cream supplemented with Cornelian cherry (*Cornus mas* L.). Paste Kafkas Univ Vet Fak Derg. (2017) 23:691–7. 10.9775/574kvfd.2016.17298

[43] Torri L Piochi M Marchiani R Zeppa G Dinnella C Monteleone E. A sensory- and consumer-based approach to optimize cheese enrichment with grape skin powders. J. Dairy Sci. (2016) 99:194–204. 10.3168/jds.2015-9922 26585476

[44] Sulejmani E Sahingil D Adnan AA. Comparative study of compositional, antioxidant capacity, ACE-inhibition activity, RP-HPLC peptide profile and volatile compounds of herbal artisanal cheeses. Int Dairy J. (2020) 111:104837. 10.1016/j.idairyj.2020.104837

[45] Lee NK Jeewanthi RKC Park EH Paik HD. Short communication: physicochemical and antioxidant properties of Cheddar-type cheese fortified with Inula britannica extract. J. Dairy Sci. (2016) 99:83–8. 10.3168/jds.2015-9935 26519976

[46] Lamothe S Langlois A Bazinet L Couillard C Britten M. Antioxidant activity and nutrient release from polyphenol-enriched cheese in a simulated gastrointestinal environment. Food Funct. (2016) 7:1634–44. 10.1039/C5FO01287B 26931486

[47] Rachtan-Janicka J Ponder A Hallmann E. The effect of organic and conventional cultivations on antioxidants content in blackcurrant (*Ribes nigrum* L) species. Appl Sci. (2021). 11:5113. 10.3390/app11115113

[48] Papoutsis K Pristijono P Golding JB Stathopoulos CE Bowyer MC Scarlett CJ. Effect of vacuum-drying, hot air-drying and freeze-drying on polyphenols and antioxidant capacity of lemon (*Citrus limon*) pomace aqueous extracts. Int J Food Sci Technol. (2017) 52:880–7. 10.1111/ijfs.13351

[49] Olszewska MA Gedas A Simões M. Antimicrobial polyphenol-rich extracts: applications and limitations in the food industry. Food Res Int. (2020) 134. 10.1016/j.foodres.2020.109214 32517896

[50] Li J Huang Q Zheng X Ge Z Lin K Zhang D. Investigation of the lactic acid bacteria in kazak cheese and their contributions to cheese fermentation. Front Microbiol. (2020) 11:1–13. 10.3389/fmicb.2020.00228 32226414 PMC7080652

[51] Carafa I Nardin T Larcher R Viola R Tuohy K Franciosi E. Identification and characterization of wild lactobacilli and pediococci from spontaneously fermented mountain cheese. Food Microbiol. (2015) 48:123–32. 10.1016/j.fm.2014.12.003 25791000

[52] Cogan TM Barbosa M Beuvier E Bianchi-Salvadori B Cocconcelli PS Fernandes I. Characterization of the lactic acid bacteria in artisanal dairy products. J Dairy Res. (1997) 64:409–21. 10.1017/S0022029997002185

[53] Wouters JTM Ayad EHE Hugenholtz J Smit G. Microbes from raw milk for fermented dairy products. Int Dairy J. (2002) 12:91–109. 10.1016/S0958-6946(01)00151-0

[54] Cipolat-Gotet C Cecchinato A De Marchi M Bittante G. Factors affecting variation of different measures of cheese yield and milk nutrient recovery from an individual model cheese-manufacturing process. J Dairy Sci. (2013) 96:7952–65. 10.3168/jds.2012-6516 24094531

[55] Everette JD Bryant QM Green AM Abbey YA Wangila GW Walker RB. Thorough study of reactivity of various compound classes toward the folin–ciocalteu reagent. J Agric Food Chem. (2010) 58:8139–44. 10.1021/jf1005935 20583841 PMC4075968

[56] Rodrigues D Santos CH Rocha-Santos TAP Gomes AM Goodfellow BJ Freitas AC. Metabolic profiling of potential probiotic or synbiotic cheeses by nuclear magnetic resonance (NMR) spectroscopy. J Agric Food Chem. (2011) 59:4955–61 10.1021/jf104605r 21443163

[57] Akoka S Barantin L Trierweiler M. Concentration measurement by proton NMR using the ERETIC. Met Analytical Chem. (1999) 71:2554–2557. 10.1021/ac981422i 21662801

[58] Hong RS Hwang KH Kim S Cho HE Lee HJ Hong JT. Survey of ERETIC2 NMR for quantification. J Korean Magnetic Resonance Soc. (2013) 17:98–104 10.6564/JKMRS.2013.17.2.098

[59] Watanabe R Sugai C Yamazaki T Matsushima R Uchida H Matsumiya M. Quantitative nuclear magnetic resonance spectroscopy based on PULCON methodology: application to quantification of invaluable marine toxin, okadaic acid. Toxins. (2016) 8:294. 10.3390/toxins8100294 27754382 PMC5086654

[60] Cullen CH Ray GJ Szabo CM. Comparison of quantitative nuclear magnetic resonance methods: internal, external, and electronic referencing. Magn Reson Chem. (2013) 51:705–13. 10.1002/mrc.4004 24002733

[61] Wishart DS Feunang YD Marcu A Guo AC Liang K Vázquez-Fresno R. HMDB 4.0: the human metabolome database for 2018. Nuc Acids Res. (2018) 46:D608–17. 10.1093/nar/gkx1089 29140435 PMC5753273

[62] Minim VPR Della Lucia SM. Grupo de foco. In:Minim VPR, editor. Anàlise sensorial: estudos com consumidores. Vicosa: Editora. UFV, Cap. 4 (2006). p. 85–109.

[63] Krzyściak P Krośniak M Gastoł M Ochońska D Krzyściak W. Antimicrobial activity of cornelian cherry (*Cornus mas* L.). Postepy Fitoterapii. (2011) 4:227–31.

[64] Milenković-Andjelković AS Andjelković MZ Radovanović AN Radovanović BC Nikolić V. (2015). Phenol composition, DPPH radical scavenging and antimicrobial activity of cornelian cherry (*Cornus mas*) fruit and leaf extracts. Hemijska Industrija. 69:331–7. 10.2298/HEMIND140216046M

[65] Jeong CH Jang CW Lee KY Kim IH Shim KH. Chemical components and anti-oxidant activities of black currant. Korean Soc Food Preserv. (2012) 19:263–70. 10.11002/kjfp.2012.19.2.26331800022

[66] Antoniewska-Krzeska A Ivanišová E Klymenko S Bieniek AA Fatrcová Šramková K Brindza J. Nutrients content and composition in different morphological parts of Cornelian cherry (*Cornus mas* L.). Agrobiodivers Improv Nutr Health Life Qua. (2022) 6. Available online at: https://agrobiodiversity.uniag.sk/scientificpapers/article/view/426

[67] Dzydzan O Bila I Kucharska AZ Brodyak I Sybirna N. Antidiabetic effects of extracts of red and yellow fruits of cornelian cherries (*Cornus mas* L) on rats with streptozotocin-induced diabetes mellitus. Food Function. (2019) 16:6459–72. 10.1039/C9FO00515C 31528975

[68] Fox PF Law J McSweeney PLH Wallace J. Biochemistry of Cheese Ripening PF Fox (Ed.), Cheese: Chemistry, Physics and Microbiology, General Aspects (2nd ed.), London: Chapman and Hall (1993). p. 415–7.

[69] Ladero V Linares DM Fernández M Alvarez MA. Real time quantitative PCR detection of histamine-producing lactic acid bacteria in cheese: relation with histamine content. Food Res Int. (2008) 41:1015–9. 10.1016/j.foodres.2008.08.001

[70] Commission Regulation (EC) No. 2073/2005 of 15 November 2005 on Microbiological Criteria for Foodstuffs. Official Journal of the European Union, Vol. 338. The European Commission (2014). p.12.

[71] Ruiz-Capillas C Herrero AM. Impact of biogenic amines on food quality and safety. Foods. (2019) 8:62. 10.3390/foods8020062 30744001 PMC6406683

[72] Cui Y Miao K Niyaphorn S Qu X. Production of gamma-aminobutyric acid from lactic acid bacteria: a systematic review. Int J Mol Sci. (2020) 21:995. 10.3390/ijms21030995 32028587 PMC7037312

[73] Barbaccia P Francesca N Gerlando RD Busetta G Moschetti G Gaglio R. Biodiversity and dairy traits of indigenous milk lactic acid bacteria grown in presence of the main grape polyphenols. FEMS Microbiol Lett. (2020) 367:fnaa066. 10.1093/femsle/fnaa066 32286619

[74] Wong CG Bottiglieri T Snead OC 3rd. GABA, gamma-hydroxybutyric acid, and neurological disease. Ann Neurol. (2003) 54:S3–12. 10.1002/ana.10696 12891648

[75] Tabasco R Sánchez-Patán F Monagas M Bartolomé B Moreno-Arribas MV Peláez C. Effect of grape polyphenols on lactic acid bacteria and bifidobacteria growth: Resistance and metabolism. Food Microbiol. (2011) 28:1345–52. 10.1016/j.fm.2011.06.005 21839384

